# Functional role of ALK-related signal cascades on modulation of epithelial-mesenchymal transition and apoptosis in uterine carcinosarcoma

**DOI:** 10.1186/s12943-017-0609-8

**Published:** 2017-02-14

**Authors:** H Inoue, M Hashimura, M Akiya, R Chiba, M Saegusa

**Affiliations:** Department of Pathology, Kitasato University School of Medicine, 1-15-1 Kitasato, Minami-ku, Sagamihara, 252-0374 Kanagawa Japan

**Keywords:** ALK, Akt, NF-κB, Twist1, EMT, Apoptosis, Uterine carcinosarcoma

## Abstract

**Background:**

Anaplastic lymphoma kinase (ALK), which is a receptor tyrosine kinase, is essentially and transiently expressed in the developing nervous system. Recently, the deregulated expression of full-length ALK has been observed in some primary solid tumors, but little is known about its involvement in the tumorigenesis of uterine carcinosarcomas (UCSs). Here we examined the functional role of the *ALK* gene in UCSs.

**Methods:**

Regulation and function of the *ALK* gene were assessed using two endometrial carcinoma cell lines. Expression of ALK and its related molecules were also investigated using clinical samples of UCSs.

**Results:**

In cell lines, *ALK* promoter activity was significantly increased by transfection of Sox11 and N-myc, which are known to contribute to neuronal properties. Cells stably overexpressing full-length ALK showed an enhancement of EMT properties mediated by TGF-β1 and HGF, along with an increase in phosphorylated (p) Akt and nuclear p65. Overexpression of p65 also led to transactivation of *Twist1* gene, known as an EMT inducer. Finally, treatment of the stable ALK-overexpressing cells with doxorubicin resulted in inhibition of apoptosis with progressive increase in the expression ratio of both pAkt and bcl2 relative to total Akt and bax, respectively. In clinical samples, strong cytoplasmic ALK immunoreactivity and mRNA signals without rearrangement or amplification of the *ALK* locus were frequently observed in UCSs, particularly in the sarcomatous components. Further, ALK IHC score was found to be positively correlated with Sox11, N-myc, Twist1, and bcl2 scores.

**Conclusion:**

ALK-related signal cascades containing Akt, NF-κB, Twist1, and bcl2 may participate in initial signaling for divergent sarcomatous differentiation driven from carcinomatous components in UCSs through induction of the EMT process and inhibition of apoptotic features.

**Electronic supplementary material:**

The online version of this article (doi:10.1186/s12943-017-0609-8) contains supplementary material, which is available to authorized users.

## Background

Uterine carcinosarcomas (UCSs), previously referred to as malignant mixed mullerian tumors, are aggressive neoplasms with biphasic growth of high-grade carcinomatous and sarcomatous elements, and only account for approximately 2–5% of all malignancies of the uterine corpus [1, 2]. The most common epithelial components are the serous type followed by endometrioid type, while the sarcomatous component is composed of homologous or heterologous tissues [3, 4]. Recent studies revealed that most, but not all, UCSs are derived from a single epithelial cell, supporting the idea that UCSs represent metaplastic carcinomas [3, 5].

The *anaplastic lymphoma kinase (ALK)* gene, located on chromosome 2p23, is highly homologous to that of *leukocyte tyrosine kinase (LTK)* and further belongs to the insulin receptor superfamily of receptor tyrosine kinases (RTK) [6–10]. Full-length ALK is specifically expressed in the developing central and peripheral nervous system during embryogenesis and is associated with the balance of cell proliferation and differentiation [11–13]. While several fusion genes involving *ALK* produced by chromosomal rearrangements have been found in a subset of lymphomas and lung carcinomas,[7, 14] recently, deregulated expression of full-length ALK has also been observed in some primary solid tumors derived from various tissues [15].

Epithelial-mesenchymal transition (EMT) plays a central role in converting both normal and neoplastic epithelial cells into derivatives with a more mesenchymal phenotype [16, 17]. A hallmark of EMT is loss of cell-cell adhesion molecules, down-regulation of epithelial differentiation markers, and transcriptional induction of mesenchymal markers [18]. Snail, Slug, and Twist, all repressors of the *E-cadherin* gene, are also involved in the process [19–22]. Interestingly, carcinosarcomas are considered to represent a true example of complete EMT [23, 24].

The oncogenic role of ALK is mediated by interactions with downstream molecules that trigger substantial intracellular signaling cascades, and is closely associated with EMT properties [25–28]. We therefore hypothesized that ALK may contribute to the determination of the phenotypic characteristics of UCS cells through regulation of its downstream transduction cascades that pertain to the EMT process. To test this, we hereby investigated the expression of ALK, as well as the profiles of its related molecules, using endometrial carcinoma (Em Ca) cell lines and clinical UCS samples.

## Methods

### Plasmids and cell lines

Full-length cDNA of human ALK, c-myc, and N-myc (Open Biosystems, Huntsville, AL, USA) were subcloned into pcDNA3.1 (Invitrogen, Carlsbad, CA, USA). The human *ALK* promoter between −2056 and +30 bp and the human *N-myc* promoter encompassing −221 to +1312 bp (where +1 represents the transcription start site) were amplified by polymerase chain reaction (PCR) and were subcloned into the pGL-3B vector (Promega, Madison, WT, USA). The human *Twist 1* promoter (GenBank accession number NG008114) between −1085 to +350 bp was also cloned using similar procedures. A series of 5’-truncated promoter constructs of *ALK* and *Twist1* genes were generated by PCR-based methods. Site-directed mutagenesis in putative E1- and E2-boxes in the *ALK* promoter region was also carried out using the PrimeS-TAR Mutagenesis Basal kit (Takara Bio, Shiga, Japan). The pGL3B-Snail (containing the −1109/+36 sequence), pGL3B-Slug (−2125/-235 bp), pcDNA3.1-Sox2, pcDNA3.1-Sox3, pcDNA3.1-Sox4, pcDNA-Sox5, pcDNA3.1-Sox6, pcDNA3.1-Sox7, pcDNA3.1-Sox9, pcDNA3.1-Sox11, pcDNA3.1-Sox17, pcDNA3.1-mouse p65, and pNF-κB were also employed as described previously [29, 30]. Two sets of short hairpin oligonucleotides directed against ALK were designed using the siDirect version 2 software. Single-stranded ALK oligonucleotides were first annealed and then cloned into *Bam*HI-*Eco*RV sites of the RNAi-Ready pSIREN-RetroQ vector (Takara, Shiga, Japan), according to the manufacturer’s instructions. The sequences of PCR primers used in this study are listed in Table 1.

**Table 1:** Primer sequences used in the study

Assay	Gene/region		Sequence
Promoter	ALK	−2056 Forward	5'-GCTCGCTAGCCTCGAACTGTGTGATGTGTTAG-3'
		−1456 Forward	5'-GCTCGCTAGCCTCGATGAGATAATTCTTTG-3'
		−956 Forward	5'-GCTCGCTAGCCTCGATGAGTTCTGTGTTGG-3'
		−416 Forward	5'-GCTCGCTAGCCTCGAAGTCGGACCCGTTTA-3'
		−146 Forward	5'-GCTCGCTAGCCTCGAAGGCCGGACTGCGTG-3'
		+30 Reverse	5'-TCTTGATATCCTCGAGTACCAGCTGCTACC-3'
	N-myc	−221Forward	5'-CTCGCTAGCCTCGCAGCAGCTTTGCAGCCTTCTC-3'
		+1312 Reverse	5'-AACCAGGTTCCCCAATCTTC-3'
	Twist 1	−1086 Forward	5'-GCGTATCCAAGCATTTGGAATTGGGG-3'
		−601Foward	5'-CCCAGGACCTCCGGGCTGGG-3'
		−101 Forward	5'-GGGGACTGGAAAGCGGAAAC-3'
		+101 Forward	5'-GCGTCCAGCCGTTGGGCGCT
		+350 Reverse	5'-CTCTCGAGCGGCGACGCGTGGCCTC-3'
Mutagenesis	ALK E-box1	Forward	5'-GCTGTATAGTGGCGGGCGCCCAGGCAG-3'
		Reverse	5'-GCCCGCCACTATACAGCTGGCTGAGCCGCGC-3'
	ALK E-box2	Forward	5'-CAGGTATAGTGCGATCCAGCGGCTCTG-3'
		Reverse	5'-GGATCGCACTATACCTGGGCGCCCGCCACTT-3'
shRNA	sh2969	Forward	5'-GATCCCGAATATTAAGCATTATCTAAAGCT
			TCCTGTCACTTTAGATAATGCTTAATATTCTTTTTTG-3'
		Reverse	5'-AATTCAAAAAAGAATATTAAGCATTATCTA
			AAGTGACAGGAAGCTTTAGATAATGCTTAATATTCGG-3'
	sh2386	Forword	5'-GATCCCGTACAAACCAGTTAATCCAGAGCT
			TCCTGTCACTCTGGATTAACTGGTTTGTACTTTTTTG-3'
		Reverse	5'-AATTCAAAAAAGTACAAACCAGTTAATCCA
			GAGTGACAGGAAGCTCTGGATTAACTGGTTTGTACGG-3'
ChIP	ALK	−126 Forword	5'-GCGGAGTTGGCTTGTGAGCC-3'
		+12 Reverse	5'-TGCTACCACCGCTGCCGCC-3'
	Twist 1	−101 Forward	5'-GGGGACTGGAAAGCGGAAAC-3'
		+62 Reverse	5'-TGCAGAGCCCGCGAGGTGT-3'
mRNA	Twist1	Forward	5'-ATGATGCAGGACGTGTCCAGC-3'
		Reverse	5'-CTAGTGGGACGCGGACATGG-3'
	N-myc	Forward	5'-TTCTCACGCTCAGGGACCACGT-3'
		Reverse	5'-GAAGCGTCTAGCAAGTCCGAGC-3'
	Slug	Forward	5'-ACGCAATCAATGTTTACTCG-3'
		Reverse	5'-TGAAGAGAAAGGTTACTGTC-3'
	Snail	Forward	5'-TGCCTCGACCACTATGCCGC-3'
		Reverse	5'-AGCATTGGCAGCGAGGCGGT-3'

The Em Ca cell lines, Ishikawa and Hec251 cells, were maintained in Eagle’s MEM with 10% bovine calf serum. The full-length ALK expression plasmid or empty vector was transfected into Hec251 cells, and the stable overexpressing clones were established as described previously [31].

### Antibodies and reagents

Anti-ALK, anti-phospho-Akt at serine (Ser) 473 (pAkt), anti-Akt, anti-Slug, anti-Snail, and anti-cleaved caspase 3 antibodies were purchased from Cell Signaling (Danvers, MA, USA). Anti-Sox11 and anti-β-actin antibodies and doxorubicin were obtained from Sigma-Aldrich Chemicals (St. Louis, MO, USA). Anti-N-myc, anti-Twist1, and anti-Histone H1 antibodies were from Abcam (Cambridge, MA, USA). Anti-NF-κB/p65, anti-p27^kip1^, and anti-bax antibodies were from BD Biosciences (San Jose, CA, USA). Anti-bcl-2 and anti-p21^waf1^ antibodies were from Dako (Glostrup, Denmark). Anti-cyclin A antibody was from Novocastra (Newcastle, UK). Recombinant human tumor necrosis factor (TNF)-α, transforming growth factor (TGF)-β1, and hepatocyte growth factor (HGF) were purchased from R&D Systems (Minneapolis, MN, USA).

### Transfection

Transfection was carried out using LipofectAMINE PLUS (Invitrogen), in duplicate or triplicate as described previously [26–28]. Luciferase activity was assayed as described previously [29–31]. The two siRNAs against NF-κB/p65 or the negative control were transfected using the siPort NeoFx transfection agent (Ambion, Austin, TX, USA), according to the manufacturers’ instructions.

### Real-time reverse-transcription (RT)-PCR

cDNA was synthesized from 2 μg of total RNA. For quantitative analysis, real-time RT-PCR was carried out using the Power SYBR Green PCR Master Mix (Applied Biosystems, Foster City, CA, USA) with specific primers (Table 1). Fluorescent signals were detected using the ABI 7500 real-time PCR system, and data were analyzed using the associated ABI 7500 System SDS software (Applied Biosystems). Analysis of the *GAPDH* gene was also applied as internal control, as described previously [29–31].

### Western blot assays

Total cellular proteins were isolated using RIPA buffer [20 mM Tris–HCl (pH7.2), 1% Nonidet p-40, 0.5% sodium deoxycholate, 0.1% sodium dodecyl sulfate]. The nuclear fraction was prepared using NE-PER Nuclear and Cytoplasmic Extraction Reagents (Pierce Biotech., Rockford, IL, USA). Aliquots of the proteins were resolved by SDS-PAGE, transferred to PVDF membranes, and probed with primary antibodies coupled to the ECL detection system (Amersham Pharmacia Biotechnology, Tokyo, Japan). The intensity of individual signals was measured using ImageJ software version 1.41 (NIH, Bethesda, MD, USA).

### Flow cytometry

Cells were fixed using 70% alcohol and stained with propidium iodide (Sigma-Aldrich) for cell cycle analysis. The prepared cells were analyzed by flow cytometry using BD FACS Calibur (BD Biosciences) and CellQuest Pro software (BD Biosciences).

### Chromatin immunoprecipitation (ChlP) assay

ChIP analysis was performed using the EpiXplore ChIP assay kit (Clontech Laboratory, Mountain View, CA, USA). Briefly, cells were cross-linked with formaldehyde after transient transfection of pcDNA3.1-mouse p65. Cell lysates were sonicated to shear DNA to lengths between 200 and 1000 bp, and then precipitated overnight using anti-NF-κB/p65 antibody or mouse IgG as negative control, along with magnetic beads. After proteinase K digestion, DNA was extracted and analyzed by PCR. ChIP analysis was conducted with a reduction in the number of cycles from 30 to 25, using four specific primer sets (Table 1).

### Immunofluorescence

Hec251 cells stably overexpressing full-length ALK were incubated with anti-ALK antibody. FITC-labeled anti-rabbit IgG (Molecular Probes, Eugene, OR, USA) was used as secondary antibody as described previously [26–28].

### Clinical cases

We reviewed cases of comprehensively staged high-grade endometrial adenocarcinomas from the patient records of Kitasato University Hospital for the period from 1997 to 2015. According to the criteria of the 2014 World Health Organization classification, [32] tumors were designated as UCS if they had evidence of both malignant epithelial (endometrioid, serous, or clear cell components) and mesenchymal (homologous or heterologous) elements. Endometrioid adenocarcinomas with spindle elements and hyalinized stroma were specifically excluded. Finally, a total of 27 UCSs were investigated (Table 2). Of these, 20 cases had endometrioid components and 7 cases contained non-endometrioid epithelial components, including serous and clear types, while 21 and 6 cases showed homologous and heterologous mesenchymal elements, respectively. All tissues were routinely fixed in 10% formalin and processed for embedding in paraffin wax. Approval for this study was given by the Ethics Committee of the Kitasato University School of Medicine (B14–35).

**Table 2:** Summary of the profiles of ALK and its related molecules in 27 uterine carcinosarcoma cases

				ALK					Sox11		N-myc		pAkt		Twist1		bcl2	
Case	Age	Histology		IHC		ISH		FISH	IHC		IHC		IHC		IHC		IHC	
No.	(year)	Ca	Sa	Ca	Sa	Ca	Sa		Ca	Sa	Ca	Sa	Ca	Sa	Ca	Sa	Ca	Sa
UCS 1	59	Non-E	Homo	0	0	1+	–	^a^	2	0	0	0	2	0	8	0	12	2
UCS 2	60	Non-E	Het (con)	0	0	–	–	^a^	^a^	^a^	0	0	^a^	^a^	^a^	^a^	^a^	^a^
UCS 3	61	E	Homo	0	0	^a^	^a^	^a^	0	0	0	0	2	0	4	0	0	0
UCS 4	81	E	Het (con)	2	0	1+	−	^a^	3	0	0	0	4	4	6	2	8	8
UCS 9	58	E	Homo	0	0	N	3+	^a^	^a^	^a^	^a^	^a^	^a^	^a^	^a^	^a^	2	4
UCS 10	85	E	Homo	0	0	3+	3+	^a^	0	2	0	0	2	6	0	9	0	0
UCS 19	59	E	Homo	0	0	−	−	^a^	0	0	0	0	4	4	3	12	0	0
UCS 12	76	E	Homo	0	0	3+	N	^a^	0	0	0	0	0	8	0	4	3	3
UCS 22	58	E	Homo	0	0	−	2+	^a^	0	0	0	0	0	0	0	0	0	0
UCS 25	74	E	Homo	0	2	1+	1+	^a^	^a^	^a^	^a^	^a^	^a^	^a^	^a^	^a^	0	0
UCS 26	78	Non-E	Het (con)	0	0	–	1+	^a^	0	0	0	0	2	2	0	0	^a^	^a^
UCS 27	65	E	Homo	0	2	1+	1+	^a^	0	0	0	0	0	2	0	6	^a^	^a^
UCS 29	73	E	Het (Rha)	0	0	3+	–	^a^	0	0	0	0	0	0	0	6	4	4
UCS 33	51	E	Homo	0	12	^a^	^a^	–	^a^	^a^	0	0	2	0	^a^	^a^	^a^	^a^
UCS 35	67	E	Homo	0	4	3+	2+	^a^	0	0	0	0	0	2	0	4	^a^	^a^
UCS 36	57	E	Homo	0	0	–	N	^a^	0	0	0	0	2	2	0	0	4	8
UCS 37	69	E	Homo	0	6	–	3+	–	0	0	0	0	0	0	0	2	0	4
UCS 38	59	Non-E	Homo	0	4	^a^	^a^	^a^	0	0	0	0	2	4	0	8	0	2
UCS 39	76	E	Homo	0	4	2+	2+	^a^	0	0	0	4	4	4	0	6	4	4
UCS 42	63	Non-E	Homo	0	0	−	−	^a^	0	0	0	0	2	6	0	2	0	0
UCS 44	50	Non-E	Homo	0	0	−	1+	^a^	0	0	0	0	2	2	0	6	0	0
UCS 45	75	E	Het (Rha)	0	0	−	−	^a^	0	0	0	0	4	0	0	4	0	8
UCS 46	68	E	Het (con)	0	0	2+	1+	^a^	4	0	2	0	6	6	0	12	8	2
UCS 47	69	Non-E	Homo	9	0	1+	1+	−	4	8	^a^	^a^	2	0	0	8	8	8
UCS 48	54	E	Homo	0	0	^a^	^a^	^a^	0	0	0	0	0	0	0	2	0	4
UCS 51	59	E	Homo	8	4	^a^	^a^	−	4	3	3	0	3	3	0	2	6	6
UCS 52	49	E	Homo	6	0	^a^	^a^	−	6	0	0	0	0	0	12	0	^a^	^a^

*Abbreviations*: *No*. number, *Ca* carcinomtous component, *Sa* sarcomatous component, IHC immunohistochemistry, *ISH in situ* hybridization, *FISH* Fluorescence *in situ* hybridization, *Non-E*non endometrioid, *E* endometrioid, *Homo* homologus, *Het* heterologous, *con* condrosarcoma, *Rha* rhabdomyosarcoma

^a^not examined; FISH -, no rearrangement ot amplification of ALK locus

### Immunohistochemistry (IHC)

IHC was performed using a combination of the microwave oven heating and polymer immunocomplex (Envision, Dako) methods. For immunohistochemical detection of ALK, the ALK iAEP kit (Nichirei Biosciences, Tokyo, Japan) was applied. Lung carcinoma tissues with ALK overexpression due to a gene abnormality were used as positive control.

For evaluation of IHC findings, scoring of nuclear or cytoplasmic immunoreactivity was performed, on the basis of the percentage of immunopositive cells and the immunointensity with multiplication of the values of the two parameters, as described previously [29–31]. ALK immunopositive cells located in the carcinomatous, sarcomatous, or both components were defined as epithelial, stromal, or mixed type, respectively.

### *In situ* hybridization (ISH)

Riboprobes for ALK containing nucleotides 3946 to 4633 of the *ALK* gene were generated by in vitro transcription using full length ALK cDNA, and ISH assays were performed using the GenPoint Tyramide Signal Amplification System (Dako) as described previously [33]. ISH signal score were classified into four levels, as follows: −, none; 1+, fewer than 10% positive cells; 2+, 10–30%; 3+, more than 30%. Samples with a score of either 1+, 2+, or 3+ were considered as positive and—was considered as negative.

### FIuorescence *in situ* hybridization (FISH)

For analysis of the *ALK* (2p23) locus, dual-color FISH studies were conducted on 10 UCS cases with strong ALK immunopositivity using the Vysis LSI ALK break-apart rearrangement probe (Abbott Molecular, Abbott Park, IL, USA), according to the manufacturer’s instructions.

### Statistics

Comparative data were analyzed using the Mann-Whitney *U*-test and the Spearman’s correlation coefficient. The cutoff for statistical significance was set as p < 0.05.

## Results

### Full-length ALK expression in UCS cases

**Fig. 1:**
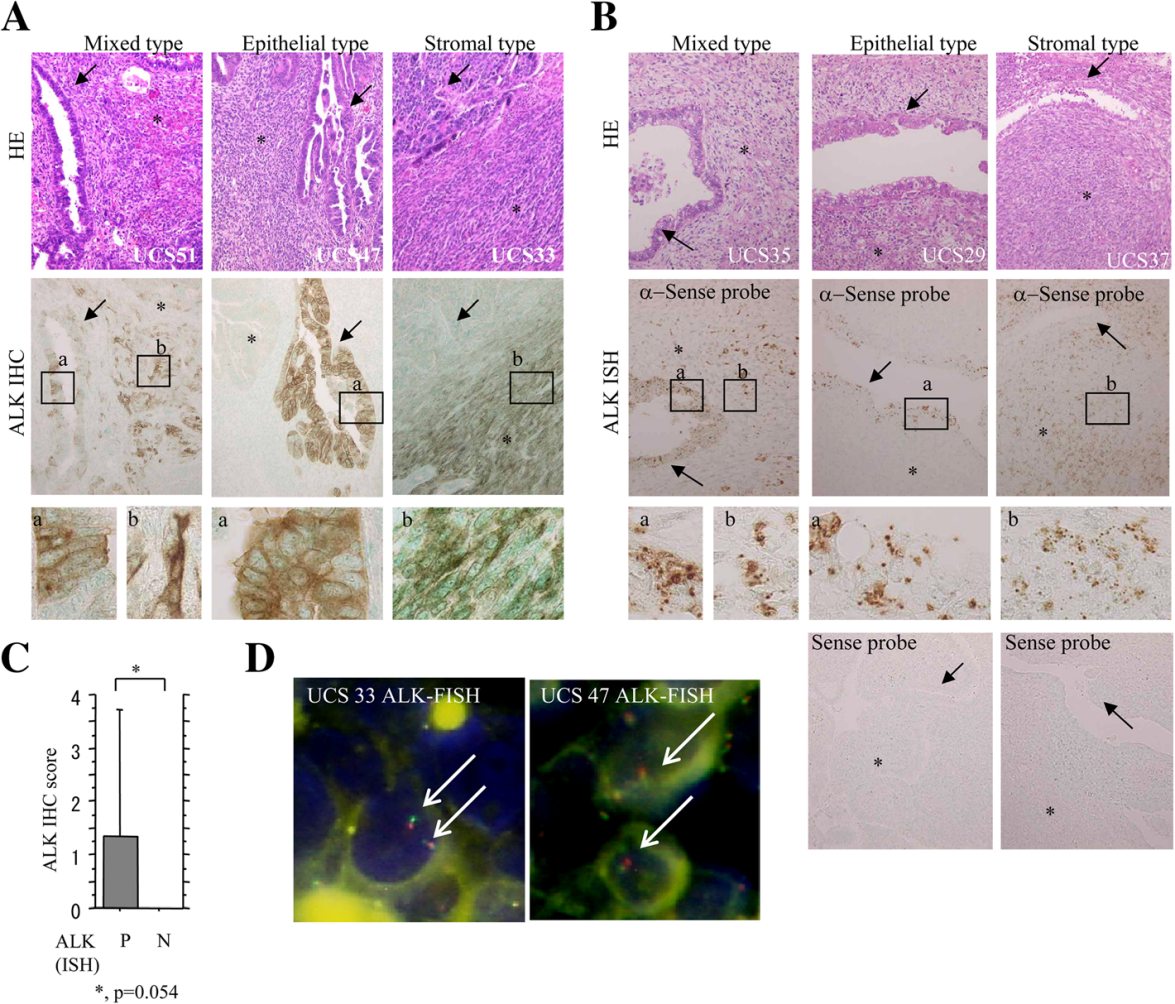
Full-length ALK expression in UCSs. **a** Staining by HE and IHC for ALK. Note the strong cytoplasmic ALK immunoreactivity in both carcinomatous (*indicated by a and arrow*) and sarcomatous components (*indicated by b and asterisk*) (*indicated by closed boxes and magnified in the insets*) in UCS51 (*mixed type*), UCS47 (*epithelial type*), and UCS33 cases (*stromal type*). Original magnification, ×100 and × 400 (inset). **b** Staining by HE and ISH for ALK mRNA. Note the mRNA signals in carcinomatous (a) and sarcomatous cells (b) (*indicated by closed boxes and magnified in the insets*) in UCS35 (*mixed type*), UCS29 (*epithelial type*), and UCS37 cases (*stromal type*). Original magnification, ×100 and × 400 (*inset*). **c** ALK IHC score in ALK mRNA-positive (*P*) and −negative (*N*) UCS cases. The data shown are means ± SDs. **d** FISH analysis of UCS33 and UCS47 cases. The interphase nuclei of both cases indicate absence of *ALK* rearrangement, in which the red and green signals remain fused (*arrows*)

Representative images of IHC findings for ALK are illustrated in Fig. 1a. Cytoplasmic immunoreaction was mainly observed in both carcinomatous and sarcomatous components of UCSs. ALK immunopositivity was evident in 11 (40.7%) of 27 UCS cases, including 3 (11.1%) of the epithelial type, 7 (25.9%) of the stromal type, and one (3.7%) of the mixed type (Table 2). In 21 UCS cases, positive signals for ALK mRNA were detected in 15 (71.4%) cases, including 4 (19%) of epithelial type, 5 (23.8%) of stromal type, and 7 (33.3%) of mixed type (Fig. 1b and Table 2). The observed ALK mRNA signals tended to be positively associated with the IHC score, although it did not reach statistical significance (Fig. 1c). Finally, FISH assay revealed no rearrangement or amplification of the *ALK* locus in 5 UCS cases with strong immunoreactivity (Fig. 1d and Table 2). These findings indicated that overexpression of full-length ALK at both mRNA and protein levels was frequently observed in UCSs.

### Activation of ALK promoter by Sox11 and N-myc

**Fig. 2:**
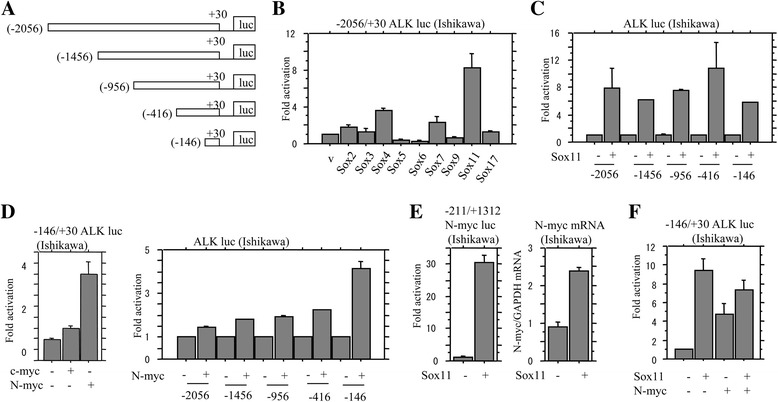
Relationship of ALK expression with Sox11 and N-myc in UCSs. **a** Various *ALK* promoter constructs used in this study. **b** Ishikawa cells were transfected with *ALK* promoter constructs, together with the indicated *Sox* genes. Relative activity was determined based on arbitrary light units of luciferase activity normalized to pRL-TK activity. The activities of the reporter plus the effector relative to that of the reporter plus empty vector are shown as means ± SDs. The experiment was performed in duplicate. **c** Ishikawa cells were transfected with various *ALK* promoter constructs, together with Sox11. **d** *Left*: the shortest ALK reporter constructs, along with either c-myc or N-myc, were transfected into Ishikawa cells. *Right*: various promoter constructs were used for evaluating transcriptional regulation of the *ALK* promoter by N-myc. **e** *Left*: Ishikawa cells were transfected with *N-myc* promoter constructs, along with Sox11. Right: analysis of mRNA levels for the *N-myc* gene by real time RT-PCR in Ishikawa cells after transfection of Sox11. **f** Ishikawa cells were transfected with *ALK* promoter constructs, together with Sox11 and N-myc

Since some *Sox* genes, as well as ALK, are essential for development of general neuronal properties, [34] we first examined the association between Sox factors and ALK expression using Ishikawa cells. Transient transfection of the longest *ALK* promoter constructs (Fig. 2a), along with nine Sox factors, revealed that Sox11, as well as Sox4 and Sox7, resulted in increased activity of the *ALK* promoter, in contrast to the inhibition by Sox5, Sox6, and Sox9 (Fig. 2b). Using a series of 5’-truncated promoter constructs (Fig. 2a), we found that deletion from −2056 to −416 bp had little effect on induction of the promoter activity by Sox11, and the shortest construct (−146/+30 bp), which lacks putative Sox-binding sites, still preserved the responsiveness to Sox11 activation (Fig. 2c).

Transient transfection of N-myc, but not c-myc, resulted in activation of the *ALK* promoter, in particular the shortest reporter constructs (−146/+30 bp) (Fig. 2d). ChIP assay also revealed that overexpression of N-myc caused its recruitment to the region from −126 to +12 bp within the *ALK* promoter (Additional file 1: Figure S1A). However, although four nucleotide alterations in E-boxes, which are binding sites for N-myc, were introduced in the shortest construct, changes in *ALK* promoter activity were relatively minor (Additional file 1: Figure S1B and C). Transfection of Sox11 also resulted in an increase in N-myc mRNA expression, along with activation of its promoter (Fig. 2e), although cooperation of Sox11 and N-myc for induction of *ALK* promoter activity was not observed (Fig. 2f). These findings suggest that both Sox11 and N-myc serve as positive transcriptional regulators for the *ALK* gene in Em Ca cells, probably through associations with the basic transcriptional machinery at the promoter.

### ALK enhances EMT phenotype by up-regulation of Twist1 through NF-κB/p65

The investigation of ALK signaling in UCSs was carried out using two Em Ca, but not UCS, cell lines, since we focused on an association between the functional roles of ALK with induction of EMT features in the carcinomatous components of UCSs. In addition, UCS cell lines are in general very rare as compared to Em Ca cells.

**Fig. 3:**
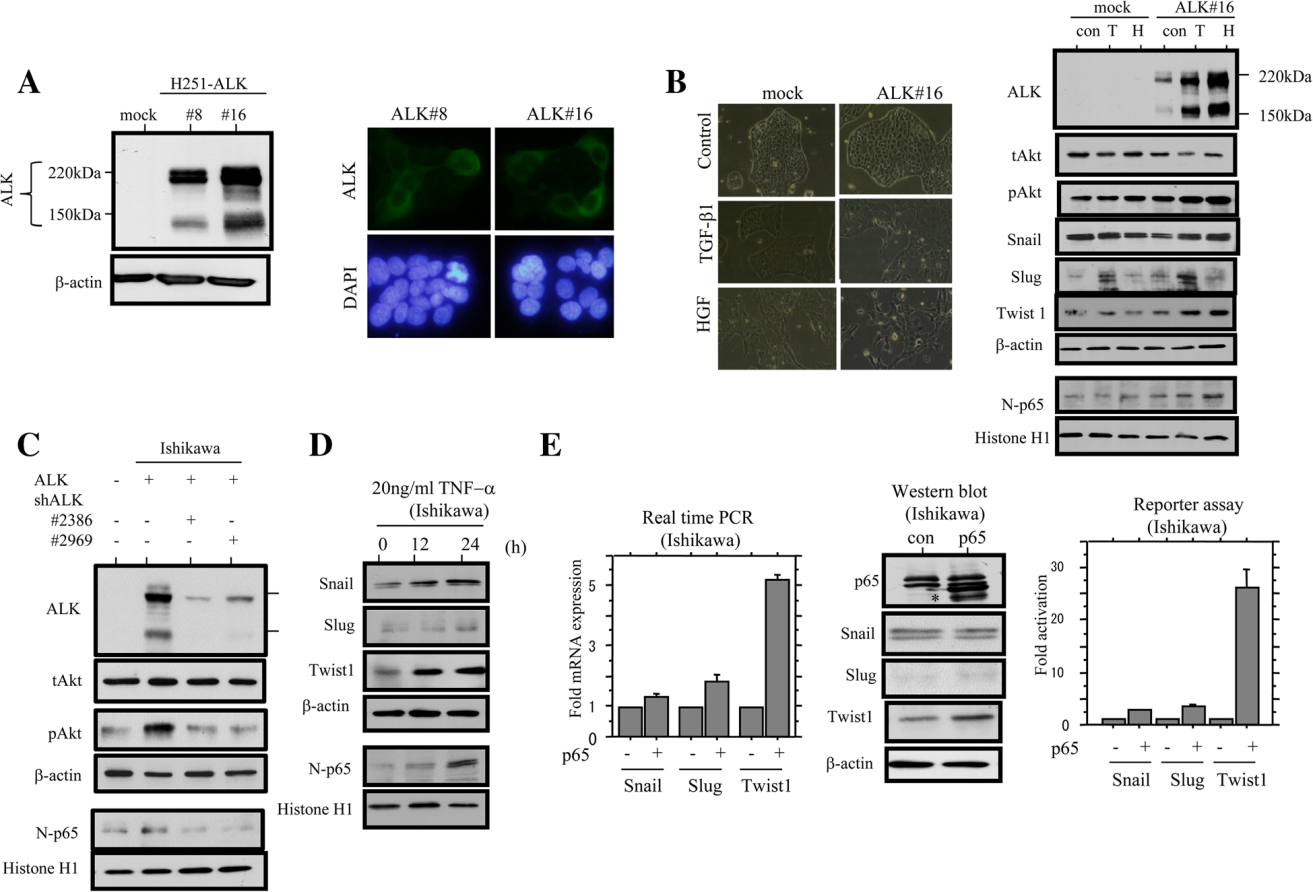
ALK/Akt/NF-κB axis in Em Ca cells. **a** Left: Hec251 cells stably overexpressing full-length ALK (H251-ALK#8 and #16, two independent clones of stable cells). Western blot assay detected exogenous ALK protein (220 kDa) and a second protein of approximately 150 kDa (*left*). *Right*: note the strong cytoplasmic ALK staining in the two independent stable cell lines. **b** *Left*: phase-contrast images of Hec251 cells stably overexpressing full-length ALK (H251-ALK#16) and the mock cells treated with 2.5 ng/ml TGF-β1 or 50 ng/ml HGF. Right: western blot analysis for the indicated proteins in ALK#16 and mock cells. con, control; T, TGF-β1; H, HGF; N, nuclear **c** Western blot analysis for the indicated proteins in Ishikawa cells after cotransfection of ALK and shRNAs against ALK. N, nuclear **d** Analysis of the indicated protein expression levels by western blot assay in Ishikawa cells after treatment with 20 ng/ml TNF-α for the time shown. N, nuclear **e** *Left*: analysis of mRNA levels for the *Snail, Slug, and Twist1* genes by real time RT-PCR in Ishikawa cells following transfection of p65. *Middle*: analysis of the indicated protein expression by western blot assay in Ishikawa cells after transfection of p65. Exogenous p65 is indicated by an asterisk in the p65 panel. con, control. *Right*: Ishikawa cells were transfected with Snail, Slug, and Twist1 reporter constructs, together with p65. Relative activity was determined based on arbitrary light units of luciferase activity normalized to pRL-TK activity. The activities of the reporter plus the effector relative to that of the reporter plus empty vector are shown as means ± SDs. The experiment was performed in duplicate

To examine whether ALK expression is closely linked to induction of EMT properties in Em Ca cells, two independent Hec251 cell lines stably overexpressing full-length ALK (H251-ALK#8 and #16) with strong cytoplasmic immunoreaction were established (Fig. 3a). These two independent stable clones showed high proliferation rates, particularly in the exponential growth phase, along with decreased amounts of p21^waf1^, but not cyclin A and p27^kip1^ (Additional file 2: Figure S2A and B). H251-ALK#16 cells treated with TGF-β1 and HGF, known as EMT inducers, demonstrated a dramatically altered morphology toward a fibroblast-like appearance after 6 days as compared to mock-treated cells, along with stabilization of exogenous full-length ALK and increased expression of pAkt, nuclear p65, as well as Twist1, but not Snail and Slug (Fig. 3b). NF-κB activity as determined by a pNF-κB reporter construct was also increased in H251-ALK#16 cells treated with TGF-β1 or HGF as compared to that of the mock cells (Additional file 2: Figure S2C). In addition, transient transfection of ALK induced increases in pAkt and nuclear p65 expression, but these effects were inhibited by cotransfection of the shRNAs against ALK in Ishikawa cells (Fig. 3c).

Given that cytokines including NF-κB effectively and reproducibly induce EMT, [35] we next examined whether p65 can affect expression of *Snail*, *Slug*, and *Twist1*, all of which are EMT-related genes. Treatment of Ishikawa cells with TNF-α resulted in dramatically increased expression of Twist1 as compared to Snail and Slug, along with stabilization of nuclear p65 (Fig. 3d). Transient transfection of p65 resulted in a considerable increase in Twist1 expression at both mRNA and protein levels, along with increased activity of its promoter. However, such associations were relatively minor for *Snail* and *Slug* (Fig. 3e).

**Fig. 4:**
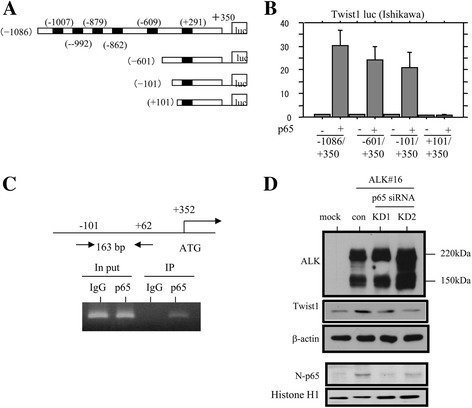
Transcriptional up-regulation of *Twist1* gene by the ALK/Akt/NF-κB axis in UCSs. **a** The *Twist1* promoter sequence containing six putative NF-κB-binding sites. **b** Various promoter constructs were used for evaluating transcriptional regulation of the *Twist1* promoter by p65. Relative activity was determined based on arbitrary light units of luciferase activity normalized to pRL-TK activity. The activities of the reporter plus the effector relative to that of the reporter plus empty vector are shown as means ± SDs. The experiment was performed in duplicate. **c** ChIP assay shows that p65 is bound to *Twist1* promoter regions. **d** Analysis of the indicated protein expression levels by western blot assay in H251-ALK#16 cells after transfection of siRNA against NF-kB/p65. KD, knockdown; con, control; N, nuclear

Next, analysis of an approximately 1000 bp fragment upstream of the transcription start site in the *Twist1* gene revealed six potential NF-κB/p65-binding elements (5’-GGRNNYYCC-3’) (Fig. 4a). Using a series of 5’-truncated promoter constructs (Fig. 4a), we found that deletion from −1086 to −101 bp had little effect on induction of the promoter activity by p65, whereas the −101/+101 bp deletion appeared to have prevented binding of p65 and reduced the promoter activity to a very low level (Fig. 4b). Similar changes in the *Twist1* promoter were also observed by TNF-α treatment (Additional file 2: Figure S2D). ChIP assay also revealed that increased amount of p65 caused its recruitment to the region of −101 to +62 bp within the promoter lacking putative NF-κB-binding sites (Fig. 4c). Finally, knockdown of endogenous p65 resulted in a decrease in Twist1 expression in H251-ALK#16 cells (Fig. 4d), although pNF-κB reporter activity was not altered by overexpression of ALK (Additional file 2: Figure S2E). These findings suggest that ALK indirectly contributes to NF-κB/p65-meditaed Twist1 expression.

### Inhibition of apoptosis by ALK through stabilization of pAkt and bcl2

**Fig. 5:**
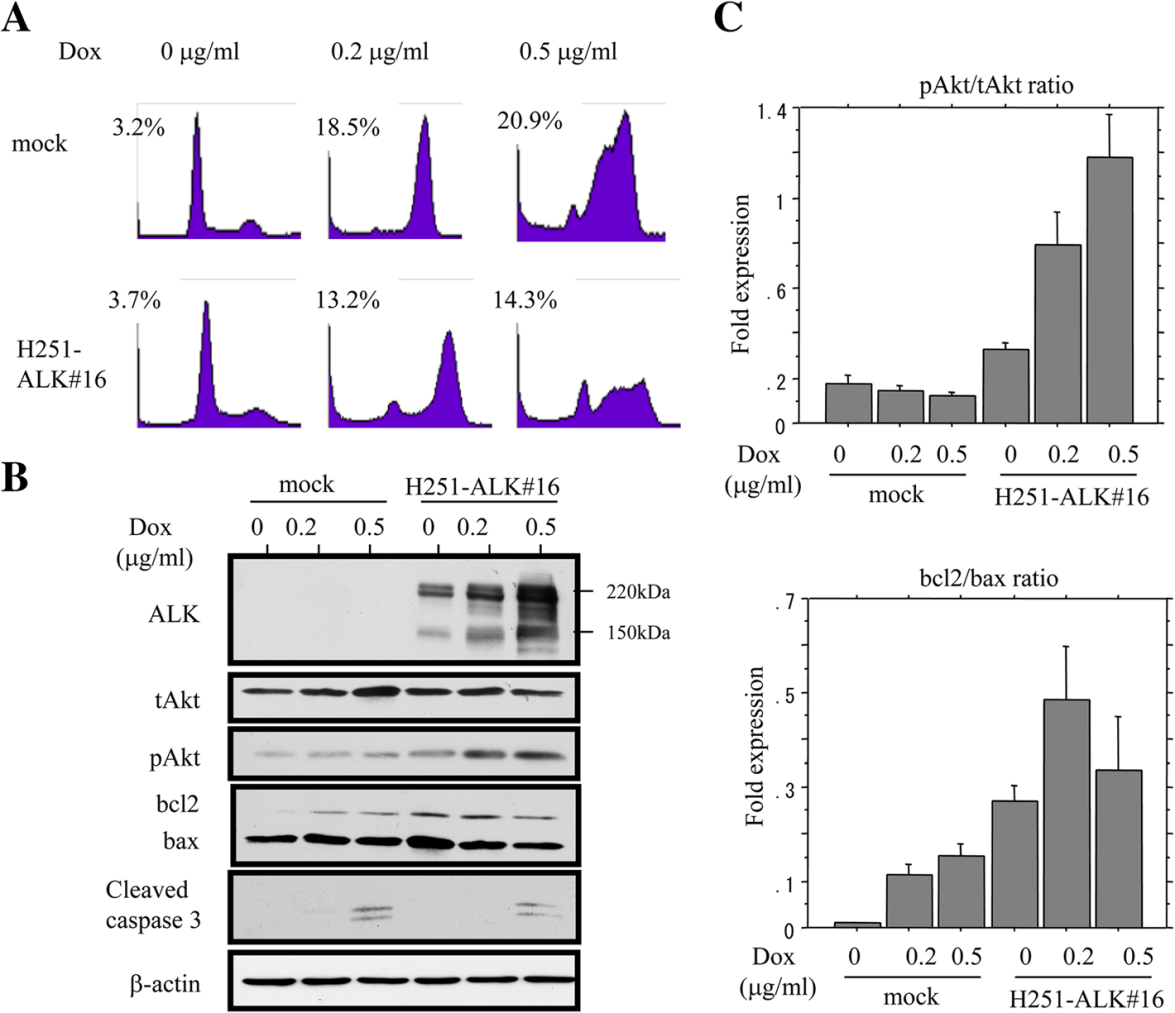
Inhibition of apoptosis by ALK/pAkt axis. **a** After treatment of Hec251 cells stably overexpressing exogenous ALK (H251-ALK#16) with doxorubicin (*Dox*) for 48 h, cells undergoing apoptosis (*sub-G1*) were detected by flow cytometry. The experiments were performed in triplicate, using independent samples. **b** Analysis of protein expression levels by western blot assay in the stable cells after treatment with Dox for 48 h. **c** Values of endogenous pAkt relative to tAkt protein (*upper*) and endogenous bcl2 relative to bax protein (*lower*) were calculated by normalization to β-actin using NIH ImageJ software

Since it is known that Akt has potential anti-apoptotic function, [36] we examined the contribution of the ALK/Akt axis to susceptibility to apoptosis in UCSs. Treatment of H251-ALK#16 with doxorubicin resulted in a reduction in the quantity of apoptotic cells as compared to mock-treated cells (Fig. 5a), along with stabilization of exogenous full-length ALK (Fig. 5b). In addition, the expression ratio of pAkt relative to total (t) Akt progressively increased in a dose-dependent manner in the doxorubicin-treated H251-ALK#16 cells as compared to the mock cells. Similar findings were also observed in the expression ratio of bcl2 relative to bax (Fig. 5c). These data indicate that overexpression of full-length ALK abrogates susceptibility to apoptosis through alteration in expression of Akt and bcl2 in Em Ca cells.

### Associations between ALK expression and the profiles of its related molecules in UCSs

**Fig. 6:**
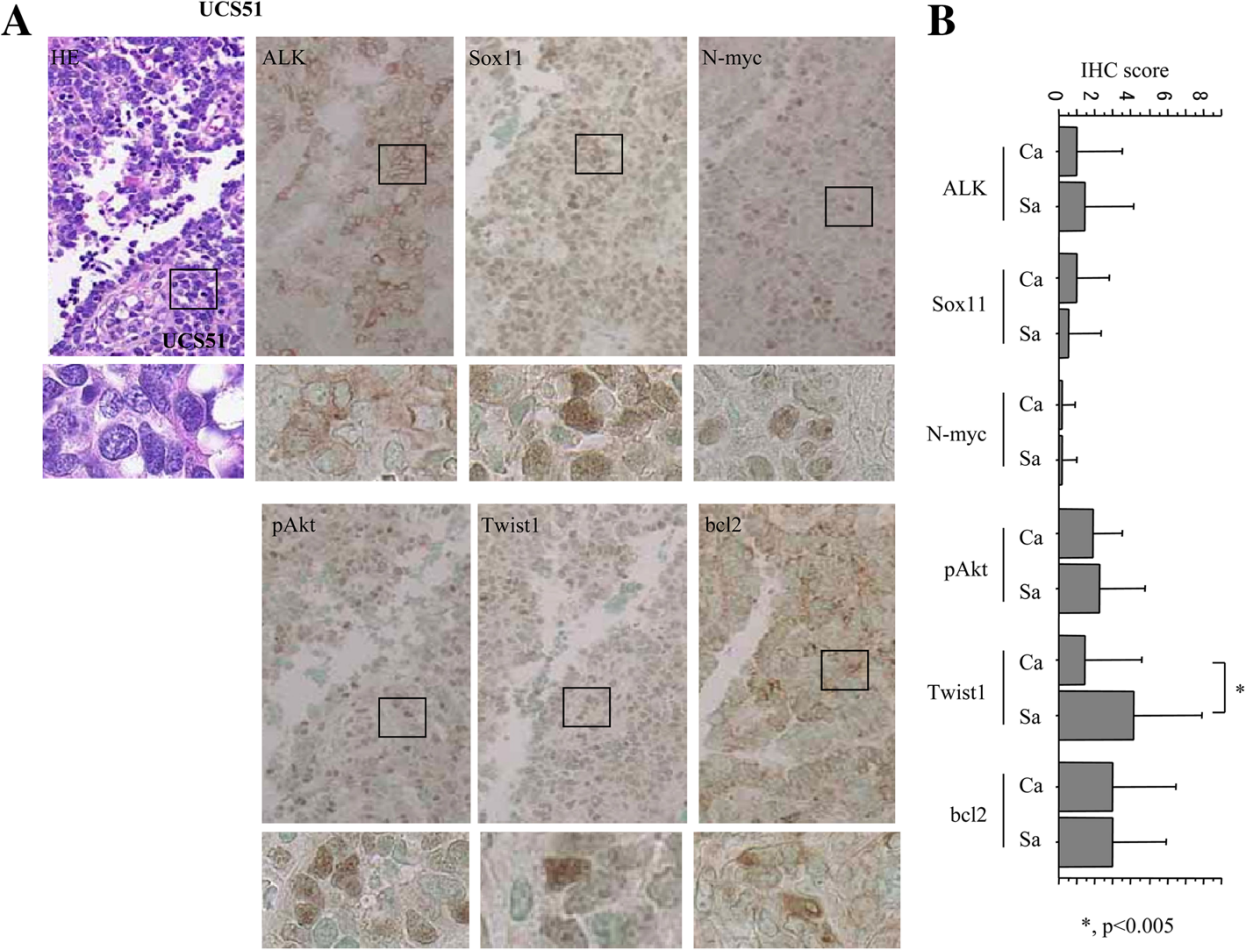
IHC findings in UCSs. **a** Staining by HE and IHC for ALK, Sox11, N-myc, pAkt, Twist1, and bcl2 in UCS51 case (*indicated by closed boxes and magnified in the insets*). Original magnification, ×100 and × 400 (*inset*). **b** IHC scores for ALK, Sox11, N-myc, pAkt, Twist1, and bcl2 in UCSs. The data shown are means ± SDs

To confirm the above findings, immunohistochemical analyses for ALK and its related molecules were carried out using clinical UCS samples. Representative IHC findings for ALK and its related molecules are illustrated in Fig. 6a. Distinct nuclear staining for Sox11, N-myc, pAkt, and Twist1 and cytoplasmic staining for bcl2 were observed in both carcinomatous and sarcomatous components, but there were no differences in the IHC scores for these markers between the two components, with the exception of Twist1 scores (Fig. 6b).

As shown in Table 3, there were positive correlations among ALK, Sox11, N-myc, and Twist1 scores in UCS tissues. The bcl2 and pAkt scores were positively correlated with ALK, Sox11, and N-myc scores, and Sox11, N-myc, and Twist1 scores, respectively. These findings supports the in vitro results that show the existence of a Sox11/N-myc/ALK axis and an association of ALK with EMT and apoptotic features through Twist1 and bcl2 expression in Ishikawa and H251-ALK#16 cells.

**Table 3:** Correlations among ALK and its related molecules in uterine carcinosarcomas

	ALK	Sox11	N-myc	pAkt	Twist1
	*r* (p)	*r* (p)	*r* (p)	*r* (p)	*r* (p)
Sox11	0.7	*	*	*	*
	(<0.0001)				
N-myc	(0.74)	0.81	*	*	*
	<0.0001	(<0.0001)			
pAkt	0.3	0.41	0.57	*	*
	(0.04)	(0.002)	(0.0001)		
Twist1	0.46	0.5	0.46	0.4	*
	(0.0002)	(0.0009)	(0.003)	(0.008)	
bcl2	0.45	0.56	0.49	0.1	0.25
	(0.005)	(0.0009)	(0.003)	(0.57)	(0.13)

*Abbreviation*: *r* Spearman’s correlation coefficient

*, not exammined

## Discussion

The present study clearly provided evidence that full-length ALK protein without chromosomal rearrangements was frequently overexpressed in UCSs, particularly in sarcomatous components. Moreover, the subcellular localization of ALK immunoreactivity was mostly cytoplasmic compartments in UCS cells, as well as in H251-ALK#16 cells, which showed increased cell proliferation as compared to the mock cells. Given the evidence that cytoplasmic localization of ALK-tyrosine kinase domain promotes cell proliferation in PC12 cells, in contrast to membrane attachment for control of neurite outgrowth and proliferation arrest, [37] it appeared that the cytoplasmic status of ALK expression may contribute to aggressive features of UCSs.

Although the ALK mRNA signals appeared to be positively associated with the immunoreactivity, some UCS cases exhibited positive ISH signals despite negative immunoreactivity. This may be due to the difference in detection sensitivity between the two assays. In addition, post-transcriptional or post-translational modification of ALK expression may also exist. In fact, discrepant results between ALK transcript and protein expression have been demonstrated in Calas (melanoma) and NCI-H69 (small cell lung carcinoma) cells [38].

Both N-myc and c-myc can induce the proximal promoter activity of the *ALK* gene through direct interaction with the E-boxes in neuroblastoma cells [39]. In this study, transfection of N-myc, but not c-myc, induced enhancement of *ALK* promoter activity in Em Ca cells, independent of E-box status. Further, transcription of both *ALK* and *N-myc* genes were positively regulated by Sox11, in line with the IHC results showing positive correlations among the three genes in USC tissues. In general, overexpression of Sox11, as well as ALK and N-myc, contributes to the activation of the expression of early genes that endow cells with general neuronal properties [34, 38, 39]. Given that UCSs potentially have neuroendocrine features, [40] it appears that activation of signal pathways containing Sox11, N-myc, and ALK may be linked to neuronal differentiation in UCSs. This conclusion is also supported by our findings showing positive correlations of bcl2 with ALK, Sox11, and N-myc scores in UCSs, since bcl2 expression is closely associated with neuroendocrine differentiation in some tumors [41, 42].

In nucleophosmin (NPM)/ALK-transformed cells, phosphatidylinositol 3-kinase (PI3K)/Akt pathway, which is emerging as a central feature of EMT, is activated by interaction of NPM-ALK with the p85 subunit of PI3K [43, 44]. In addition, activation of NF-κB signaling through phosphorylation of IκB by Akt also serves as a key factor for the process by regulating the expression of EMT master-switch transcription factors [35, 45]. In this study, H251-ALK#16 cells with stable overexpression of ALK exhibited an enhancement of EMT properties in response to treatment with EMT inducers, along with an increase in endogenous pAkt and nuclear p65. Increased pAkt and nuclear p65 expression mediated by transient transfection of ALK was abrogated by cotransfection of the shRNAs against ALK in Ishikawa cells.

Several lines of evidence from our present study support the conclusion that Twist1 expression is under the transcriptional control of p65 : i) rapid induction of Twist1 expression by treatment of cells with TNF-α; ii) up-regulation of Twist1 expression at both mRNA and protein levels by transfection of p65 in Ishikawa cells; iii) activation of the *Twist1* promoter by p65 at the proximal region (−101 to +62 bp), which is independent of NF-κB-binding sites, suggesting its association with the basic transcriptional machinery at the promoter; iv) decreased Twist1 expression by knockdown of endogenous p65 in H251-ALK#16 cells; v) and significant positive correlation of Twist1 score with both ALK and pAkt IHC scores in clinical UCS samples. Although we could not demonstrate immunoreactivity for phosphorylated NF-κB/p65 (pp65) in UCS tissues, because of a lack of the available antibody, it appears that the ALK-mediated Akt/NF-κB/Twist1 pathway may participate in an initial stage that regulates morphological alterations toward the sarcomatous phenotype in UCSs, since induction of Akt was found to activate NF-κB/p65-dependent transcription, probably through repression of IκBα expression [46].

Another interesting finding in this study was that H251-ALK#16 cells treated with doxorubicin showed a decrease in the number of apoptotic cells, along with stabilization of exogenous ALK and increased endogenous pAkt and bcl2 expression. Given the fact that Akt itself is able to prevent caspase activation by maintaining mitochondrial integrity through regulation of the bcl2 family, [36, 47] it is likely that the ALK/Akt/bcl2 axis may act as a modulator of apoptosis in UCSs.

## Conclusion

**Fig. 7:**
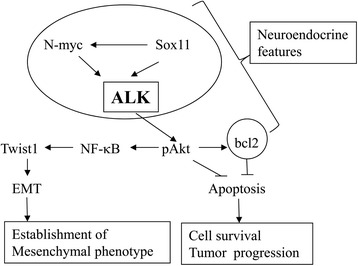
Schematic representation of ALK signal networks in modulation of apoptosis and EMT properties in UCSs

Our observations suggest some novel functional roles of ALK in UCSs (Fig. 7). Overexpression of Sox11 and N-myc causes transcriptional up-regulation of the *ALK* gene, which may be associated with the promotion of neuroendocrine features in UCS cells. The increased ALK expression triggers activation of downstream transduction cascades containing Akt, NF-κB, Twist1, and bcl2, resulting in divergent sarcomatous differentiation driven from carcinomatous components in UCSs through induction of the EMT process and inhibition of apoptosis. The conclusion is supported by further investigation using endometrial carcinoma cell lines with overexpression of endogenous full-length ALK, since carcinoma cell lines that naturally harbor full-length ALK are in general extremely rare.

## Additional files

Additional file 1: Figure S1. (A) ChIP assay shows that N-myc is bound to the proximal region (−126 to +12 bp) of the *ALK* promoter. (B) The *ALK* promoter sequence containing two putative E-boxes (E1 and E2). (C) Various promoter constructs were used for evaluating transcriptional regulation of the *ALK* promoter by N-myc. Relative activity was determined based on arbitrary light units of luciferase activity normalized to pRL-TK activity. The activities of the reporter plus the effector relative to that of the reporter plus empty vector are shown as means ± SDs. The experiment was performed in duplicate. (TIF 725 kb)
Additional file 2: Figure S2. (A) Two independent Hec251 cell lines stably overexpressing ALK (H251-ALK#8 and #16) and mock cells were seeded at low density and monitored for growth. The cell numbers presented are means ± SDs. P0, P3, P5, and P7: 0, 3,5, and 7 days after passage. (B) Western blot analysis of expression of cyclin A, p21^waf1^, and p27^kip1^ at P6 of cell growth in stable ALK-overexpressing cell lines. (C) The pNF-κB reporter construct was transfected into H251-ALK#16 cells treated with 2.5 ng/ml TGF-β1 or 50 ng/ml HGF for 48 h. Relative activity was determined based on arbitrary light units of luciferase activity normalized to pRL-TK activity. The activities of the reporter plus the effector relative to that of the reporter plus empty vector are shown as means ± SDs. The experiment was performed in duplicate. (D) Various promoter constructs were used for evaluating transcriptional regulation of the *ALK* promoter by TNF-α. (E) The pNF-κB reporter construct, together with the ALK expression vector, were transfected into Ishikawa cells. (TIF 843 kb)

## Abbreviations

ALK: Anaplastic lymphoma kinase
ChIP: Chromatin immunoprecipitation
EMT: Epithelial-mesenchymal transition
FISH: Fluorescence *in situ* hybridization
HGF: Hepatocyte growth factor
IHC: Immunohistochemistry
ISH: *In situ* hybridization
TGF: Transforming growth factor
TNF: Tumor necrosis factor
UCS: Uterine carcinosarcoma
