# Supplementary figures and images for: Functional role of ALK-related signal cascades on modulation of epithelial-mesenchymal transition and apoptosis in uterine carcinosarcoma
Source: Mol Cancer. 2017 Feb 14;16:37. doi: 10.1186/s12943-017-0609-8 (PMC5307825; doi:10.1186/s12943-017-0609-8)

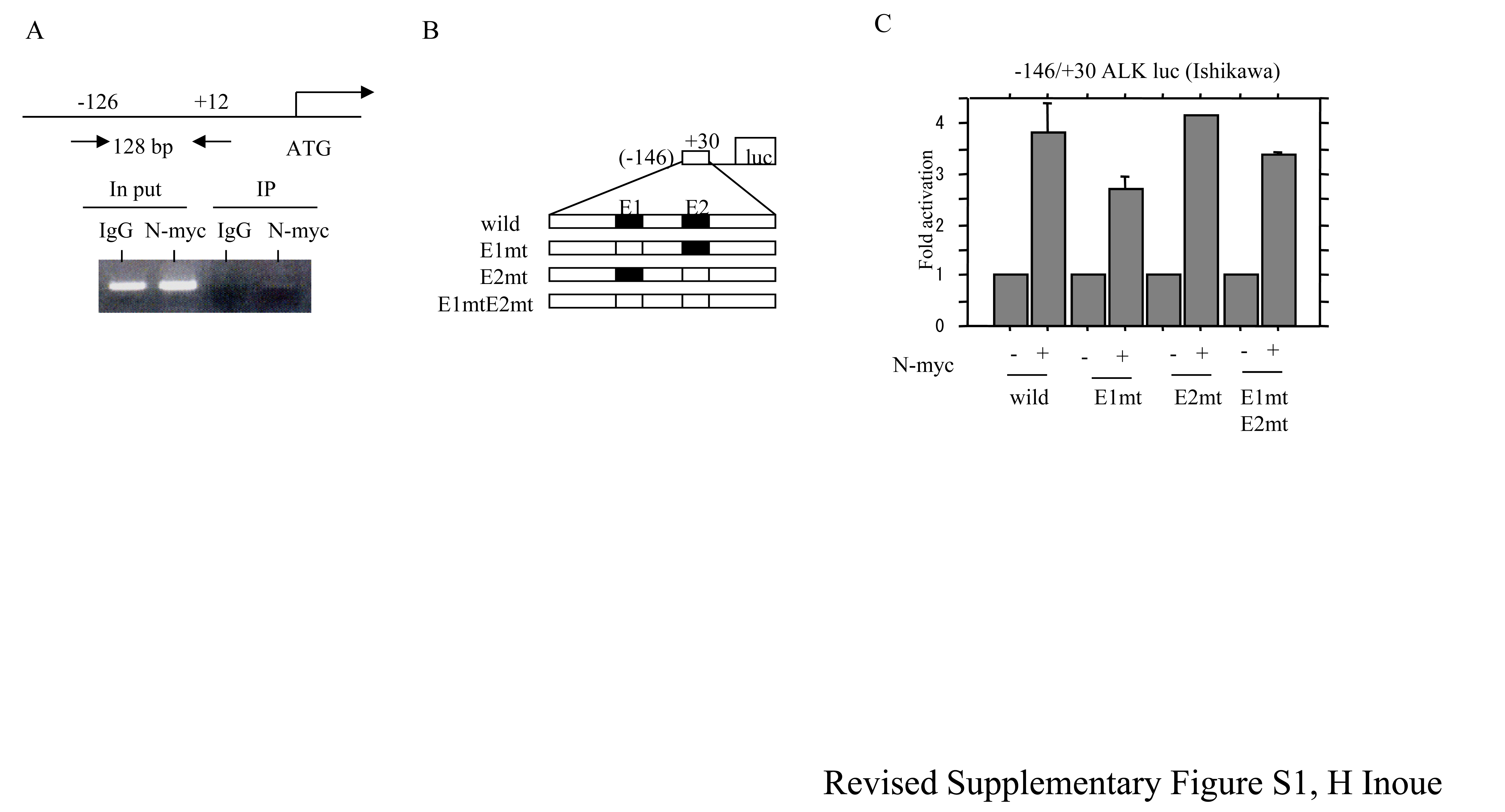

Supplement: Additional file 1: Figure S1. — (A) ChIP assay shows that N-myc is bound to the proximal region (−126 to +12 bp) of the ALK promoter. (B) The ALK promoter sequence containing two putative E-boxes (E1 and E2). (C) Various promoter constructs were used for evaluating transcriptional regulation of the ALK promoter by N-myc. Relative activity was determined based on arbitrary light units of luciferase activity normalized to pRL-TK activity. The activities of the reporter plus the effector relative to that of the reporter plus empty vector are shown as means ± SDs. The experiment was performed in duplicate. (TIF 725 kb) [file 12943_2017_609_MOESM1_ESM.tif]

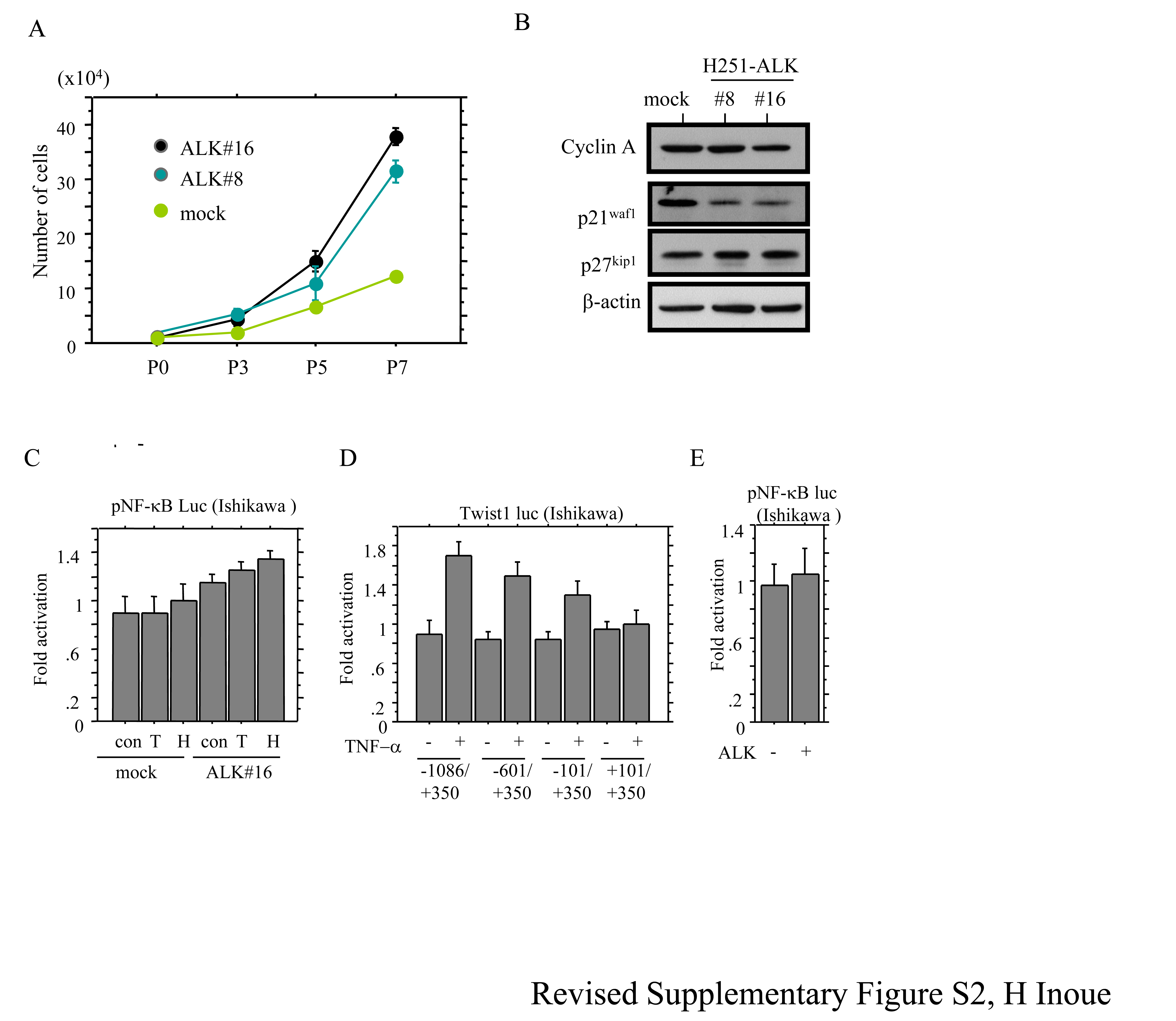

Supplement: Additional file 2: Figure S2. — (A) Two independent Hec251 cell lines stably overexpressing ALK (H251-ALK#8 and #16) and mock cells were seeded at low density and monitored for growth. The cell numbers presented are means ± SDs. P0, P3, P5, and P7: 0, 3,5, and 7 days after passage. (B) Western blot analysis of expression of cyclin A, p21waf1, and p27kip1 at P6 of cell growth in stable ALK-overexpressing cell lines. (C) The pNF-κB reporter construct was transfected into H251-ALK#16 cells treated with 2.5 ng/ml TGF-β1 or 50 ng/ml HGF for 48 h. Relative activity was determined based on arbitrary light units of luciferase activity normalized to pRL-TK activity. The activities of the reporter plus the effector relative to that of the reporter plus empty vector are shown as means ± SDs. The experiment was performed in duplicate. (D) Various promoter constructs were used for evaluating transcriptional regulation of the ALK promoter by TNF-α. (E) The pNF-κB reporter construct, together with the ALK expression vector, were transfected into Ishikawa cells. (TIF 843 kb) [file 12943_2017_609_MOESM2_ESM.tif]
